# Real Case Study of 600 m^3^ Bubble Column Fermentations: Spatially Resolved Simulations Unveil Optimization Potentials for l‐Phenylalanine Production With *Escherichia coli*

**DOI:** 10.1002/bit.28869

**Published:** 2024-10-25

**Authors:** Yannic Mast, Adel Ghaderi, Ralf Takors

**Affiliations:** ^1^ Institute of Biochemical Engineering University of Stuttgart Stuttgart Germany; ^2^ Manus Bio Augusta Georgia USA

**Keywords:** bioreactor, bubble column, computational fluid dynamics (CFD), Euler‐Lagrange approach (EL), fed batch, fermentation, kinetic model, lattice boltzmann simulation (LBM)

## Abstract

Large‐scale fermentations (»100 m³) often encounter concentration gradients which may significantly affect microbial activities and production performance. Reliably investigating such scenarios in silico would allow to optimize bioproduction. But related simulations are very rare in particular for large bubble columns. Here, we pioneer the spatially resolved investigation of a 600 m³ bubble column operating for *Escherichia coli* based l‐phenylalanine fed‐batch production. Microbial kinetics are derived from experimental data. Advanced Euler‐Lagrange (EL) computational fluid dynamics (CFD) simulations are applied to track individual bubble dynamics that result from a recently developed bubble breakage model. Thereon, the complex nonlinear characteristics of hydrodynamics, mass transfer, and microbial activities are simulated for large scale and compared with real data. As a key characteristic, zones for upriser, downcomer, and circulation cells were identified that dominate mixing and mass transfer. This results in complex gradients of glucose, dissolved oxygen, and microbial rates dividing the bioreactor into sections. Consequently, alternate feed designs are evaluated splitting real feed rates in two feeds at different locations. The opposite reversed installation of feed spots and spargers improved the product synthesis by 6.24% while alternate scenarios increased the growth rate by 11.05%. The results demonstrate how sophisticated, spatially resolved simulations of hydrodynamics, mass transfer, and microbial kinetics help to optimize bioreactors in silico.

**Abbreviations**
BSD: bubble size distribution
CFD: computer fluid dynamic
DO: dissolved oxygen
EE: Euler‐Euler approach
EL: Euler‐Lagrange approach
LBM: lattice Boltzmann method
LES: large Eddy simulation
l‐Phe: l‐phenylalanine
PBM: population balance model
*We*
: *Weber* number
*We*_*crit*_
: critical *Weber* number

## 1 Introduction

The production of (bio‐) chemicals at industrial scale requires advanced methodologies to meet the demanding market needs. To gain benefit from the economy‐of‐scale principle, large volume tanks turned out as a pivotal component for fulfilling the economic challenges in particular for commodities. For manufacturing these “large market/low margin products” such as ethanol, citric acid, and amino acids, bioreactors tailored for industrial production proved indispensable. By scaling up operations, these bioreactors contribute to cost savings while increasing the output of the desired product in a single production cycle (Sanford et al. 2016).

However, significant issues arise while operating industrial scale bioreactors. Achieving adequate mixing and efficient mass transfer is intricate due to complex flow structures inherent in multiphase systems (Wadaugsorn et al. 2016). As a consequence, concentration gradients, particularly concerning factors such as oxygen and substrate, can significantly induce stress responses in cells, thereby leading to decreased production yields (Neubauer, Häggström, and Enfors 1995; Löffler et al. 2016; Heins and Weuster‐Botz 2018). Conducting experiments on a large scale is often associated with substantial expenses and time constraints (Bisgaard et al. 2022). Modeling, on the other hand, serves as a cost‐effective substitute, facilitating swift scenario exploration, effective design adaptations, and concurrent variable adjustments to attain optimal conditions.

However, modeling industrial‐scale bioreactor performance is challenging. On the one hand, cutting‐edge modeling problems need to be solved (Siebler, Lapin, and Takors 2020). On the other hand, the scarcity of real‐world, large‐scale data for model validation poses a significant hurdle. Whereas bioreactors have been the subject of extensive research, the majority of existing literature focuses on smaller‐scale equipment. There is only a limited number of studies conducted with industrial‐sized reactors (Nauha et al. 2018; Siebler et al. 2019; Bisgaard et al. 2022; Puiman et al. 2023). This is primarily due to the formidable computational demands imposed by the development of high‐fidelity bioreactor models which necessitate extensive computing resources and simulation time to capture the highly transient and nonlinear dynamics. Consequently, these simulations heavily rely on substantial simplifications, such as one‐dimensional space solutions, time‐averaged turbulence models, or Euler‐Euler (EE) multiphase treatments that amalgamate both phases into a single continuum (Haringa et al. 2017; Chen et al. 2018; Siebler, Lapin, and Takors 2020; Ngu, Morchain, and Cockx 2022). These constraints hinder the attainment of intricate insights into the genuine hydrodynamic behavior and its far‐reaching consequences on the biological cell.

In recent years, a significant shift occurred toward enhanced use of computational fluid dynamic (CFD) simulations with the Lattice Boltzmann Method (LBM) for bioreactor simulations (Kuschel et al. 2021; Farsani et al. 2022; Gaugler et al. 2023). The approach has gained momentum thanks to advances in computing technology by parallelizing computations on graphic units. Consequently, LBM has enabled the efficient simulation of large‐scale reactors within a reasonable timeframe. The enhanced computational power enables the use of advanced Large Eddy Simulation (LES) for turbulence analysis without the reliance on time‐averaged simplifications. Furthermore, adapting the detailed analysis of Euler‐Lagrange (EL) frameworks has become a promising option to track individual bubbles with facilitated means. Introducing the bubble‐centric perspective provides a more precise locally resolved examination of multiphase interactions (Hoppe and Breuer 2020). So far, such techniques were applied for pilot or medium‐sized bioreactor simulations (Kuschel et al. 2021; Farsani et al. 2022; Gaugler et al. 2023).

This paper pioneers the rigorous use of LES and EL simulations for a real (extremely) large bioreactor providing unique insights into the intricate flow structures. The high‐resolution analysis of the dispersed phase resulted in the unprecedented simulation of bubble‐fluid interactions under real operating conditions. Benefitting from GPU‐accelerated parallelization, > 18 billion bubble‐liquid interactions were analyzed in parallel investigating the large‐scale industrial bioreactor setting under real fermentation conditions. By tracking each bubble's trajectory and considering acting forces, the EL approach provides valuable insights into bubble characteristics, breakup, and dispersion. The knowledge deems to be essential for predicting turbulent and complex flow conditions as a prerequisite for bubble‐based mixing and mass transfer. Thereon, microbial kinetics can be embedded, as it will be shown in this study. The current research builds on recent advancements in comprehending bubble dynamics (Mast and Takors 2023b) and their successful application for EL simulations (Mast and Takors 2023a).

Stirred tanks have been the conventional choice as bioreactors (Lübbert 2000). However, bubble columns offer superior gas‐liquid mass transfer, reduced shear stress for delicate microorganisms, and simpler, low‐maintenance designs with lower energy consumption (Lübbert 2000; Besagni, Inzoli, and Ziegenhein 2018). These advantages make them particularly intriguing for large scale applications of several hundred cubic meters. Biotech applications are found for the production of bakers’ yeast, citric acid, and 1,3‐propanediol (Attfield 1997; Berovic and Legisa 2007; McClure et al. 2015; Bisgaard et al. 2022). However, except for the case of 1,3 propanediol production, details about related design and operational knowhow are kept company‐owned, which renders independent simulation studies very challenging. On contrary, this contribution presents a real case simulation study analyzing an established large‐volume bubble column bioreactor. Details about geometry and operational design will be disclosed.

Furthermore, bioreactor and fed‐batch process data will be linked with microbial kinetics for the production of l‐phenylalanine (l‐Phe) via recombinant *Escherichia coli*. As a vital aromatic amino acid, l‐Phe was produced in said bioreactor as a precursor which, after being methylated, was condensated with aspartic acid to the non‐saccharide sweetener Aspartame. As such, the current modeling approach follows the promising strategy to investigate gradients in large‐scale bioreactors by linking sophisticated hydrodynamic and mass transfer models with microbial kinetics (Larsson et al. 1996; Morchain, Gabelle, and Cockx 2013).

Whereas extensive statistical evaluations have addressed bacterial lifelines and their influence on population heterogeneity (Haringa et al. 2016; Siebler et al. 2019; Nadal‐Rey et al. 2023; Puiman et al. 2023), this study investigates mass transfer characteristics, hydrodynamic phenomena, guide tube dependencies, and the crucial influence of the number and positioning of feeding points in the fermentation process, providing deeper insights for the design and operation of airlift reactors. The intricate relationships between these factors and operational variables are critical to understanding and optimizing process performance.

## 2 Materials and Methods

### 2.1 Bioreactor and Fermentation

**Figure 1:**
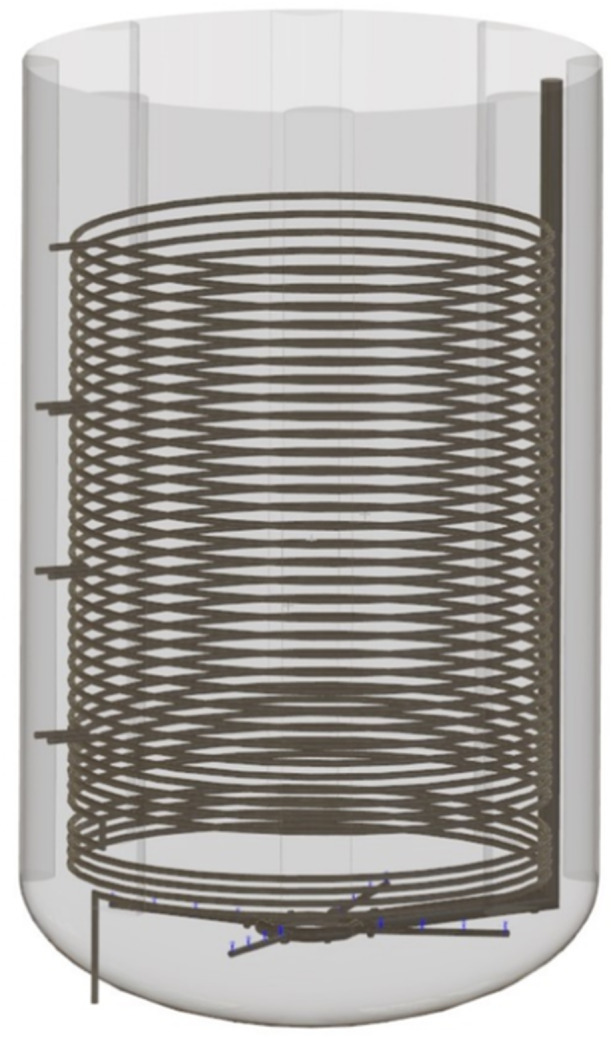
Illustration of the 600 m³ bubble column reactor geometry, featuring a four‐arm sparger and a centrally positioned heat exchange coil. The reactor dimensions (height x diameter) measure (13.06 × 7.92) m.

Operational data were obtained from the fed‐batch l‐Phe production in a bubble column (Manus Bio Inc 1762 Lovers Ln, Augusta, GA 30901, US) with 7.92 m of diameter and 13.06 m of maximum filling height resulting in 598 m^3^ of maximum filling volume. The centered, internal heat exchange coil with 6.55 m diameter and 9 m height acts as an internal expansion joint that enables a large recirculation flow. The heat exchanger coil is comprised of a tube with a diameter of 0.1 m. The tube is configured with 37 helical turns and positioned 0.74 m from the wall with a vertical clearance of 2.3 m from the top. At the base of the reactor, the four‐armed sparger is placed to inject air through 48 openings, each with 6.35 mm of diameter. Inside the bioreactor 8 vertical baffles are mounted with rounded shape (0.96 m of diameter) that are equally distributed at the inner wall. The entire reactor geometry is shown in Figure 1.

l‐Phe was produced with recombinant *E. coli* utilizing glucose in a fed‐batch fermentation. The glucose feed contained 1 kg L^−1^ and was injected at the surface of the liquid column close to the edge of the reactor. The non‐gassed liquid start volume of the fermentation vessel was 370 m³. The fed‐batch process lasted until 40 h reaching approximately 520 m³ of filling volume. Starting with the initial biomass concentration of approximately 0.5 g L^−1^, biomass growth led to 20 g L^−1^ after 24 h. Fermentation temperature was controlled at 32°C. Glucose and air supply were monitored and dynamically adjusted.

### 2.2 Simulation Setup

The CFD simulations were performed using the LBM‐based solver M‐Star 3.4.60. The standard LBM equation involves gathering a group of molecules into a probability density function called f. This function is distinct and changes over time for each numerical grid point in space. Each grid point is uniquely characterized by a probability density function f, which serves as a representation of fictitious parcels. Given in a large quantity, these fictitious parcels can effectively capture and represent the flow behavior. The LBM algorithm can be summarized in two consecutives steps: In the streaming step, as depicted in Equation (1) on the left side, the distribution function moves and thereby exchanges information with adjacent neighbor nodes with the particle velocity $\vec{v}$. In the subsequent collision step, shown on the right side in Equation (1), the fluid undergoes a relaxation process, gradually approaching a state of local equilibrium in dependency of the collision operator Q(f,f).

(1) $$\frac{\partial f}{\partial t}+\vec{v}\nabla f=Q(f,f).$$

The connection between the particle distribution function and macroscopic fluid parameters results in recovering the Navier‐Stokes equations. Using LBM as a CFD solver facilitates the straightforward parallelization, allowing for accelerated calculations on computer graphic units.

To model turbulence, LES was employed, incorporating a sub‐grid closure model based on (Smagorinsky 1963). The Smagorinsky coefficient was set to 0.1 based on experimental observations reported in (Yu, Girimaji, and Luo 2005). The quadratic uniformly distributed mesh used in the study had a grid point spacing of 1.66 cm, resulting in a total of 480 grid points along the diameter of the reactor. The grid spacing remained constant, resulting in a total grid point count of 180 million for the entire reactor. Mesh density has been chosen to keep lattice density below 1% with a time step size of 8.8∙10^−5 ^s. A mesh independence study is shown in Appendix A. With the additional memory requirements for the bubbles, the setting with the two NVIDIA RTX A6000 graphics chips, each equipped with 48GB of graphics memory, reached maximum capacity. Simulating snapshots between 400 and 600 s required up to 3 weeks. To inject air into the reactor, three neighboring air inlets were lumped together thereby reducing the number of sparger locations to 16. The gas phase was implemented as dispersed bubbles moving through the fluid. Bubbles were tracked by solving their Newtonian equations of motion in the EL framework. To manage the computational workload, one out of every 500 bubbles was explicitly tracked, and the impacts of bubble‐liquid interaction were accordingly multiplied by 500 for each bubble. Nevertheless, the equation of motion was always solved in parallel and explicitly for a minimum of 10^7^ bubbles.

Fluid and gas phases are linked via cubic correlations. The sum of all fluid forces acting opposite to the rising bubbles represents this interaction at each voxel. The equation for a drag coefficient was taken from (Brown and Lawler 2003). This choice was motivated by the observation that the gas phase achieved a stable pseudo‐steady state rather quickly. The lift coefficient is determined according to (Saffman 1965). As an additional bubble force, the virtual mass force was taken into account using a correlation proposed by (Odar and Hamilton 1964). The selection of lift and virtual mass force significantly impacted the horizontal stability of the bubbles, as dictated by two prevalent models characterized by a straightforward equation. The used equations for lift and virtual mass force are the default models in M‐Star. The adoption of the lift and virtual mass models resulted in best numerical stability in horizontal direction compared to other approaches. All air bubbles were injected with a diameter of 10 mm. The Sauter diameter is a specific type of mean diameter, used for evaluation within this study, calculated based on the ratio of the volume‐to‐surface area of the particles to their total surface area (as defined by Equation 2).

(2) $$d_{32}=\frac{\sum_{i=1}^{\infty }d_{i}^{3}}{\sum_{i=1}^{\infty }d_{i}^{2}}.$$

Bubble breakage was modeled according to the recently proposed approach of (Mast and Takors 2023a). Therein, a critical Weber number (*We*_*crit*_) is introduced. Upon surpassing this threshold, bisectional bubble breakup occurs leading to the formation of daughter bubbles with M‐shaped daughter size distribution. The *We* number depends on the liquid density *ρ*, surface tension σ, turbulent dissipation rate *ε*, and bubble diameter *d* according to:

(3) $$\mathrm{We}=\frac{\rho_{l}\varepsilon^{\frac{2}{3}}d^{\frac{5}{3}}}{\sigma }.$$

It was found that bubble breakage occurs if $\mathrm{We}>(We_{\mathrm{crit}}=6.1)$ is exceeded. The finding reflects that a minimum of turbulent dissipation energy must be outgone to initiate bubble breakage. Notably, the threshold of local energy dissipation depends on the bubble diameter and increases for smaller bubbles. To prevent nonrealistic breaking cascades, the minimum time between two consecutive breaks is limited to 30 ms as proposed by (Mast and Takors 2023a).

Noteworthy, the current simulation study does not consider bubble coalescence as salty medium composition should prevent bubble coalescence severely. Instead, bubble breakage should dominate population kinetics because of high air flows, high power inputs, and shortened bubble residence times that reflect accelerated fluid motion.

Several correlations have been published to describe the mass transfer from the gas into the liquid. The approach of (Frossling 1938) basically reflects the mindset of the film theory (Lewis and Whitman 1924). In essence, stagnant films surrounding bubbles are assumed which were questioned by the supporters of the penetration theory (see below). However, given the size of the current bubble column and the relatively long residence time of the cells the authors decided to check the applicability of the ‘film‐theory’ approach for the current scenario. Therein, the volumetric mass transfer coefficient *k*_*L*_ depends on the kinematic viscosity ν, bubble diameter d, slip velocity $u_{\mathrm{slip}}$ and on the diffusion coefficient $D_{O_{2},L}$.

(4) $$k_{L}=0.6∙\nu^{-\frac{1}{6}}∙D_{O_{2},L}^{2/3}∙\sqrt{\frac{u_{\mathrm{slip}}}{d}}.$$

For questioning the assumption of stagnant films surrounding bubbles, the well‐known penetration theory was alternatively considered. Probably the most used equation was proposed by (Higbie 1935) and is based on the so‐called penetration model.

(5) $$k_{L}=2\sqrt{\frac{u_{\mathrm{slip}}\;D_{O_{2},L}}{\pi \;d}}.$$

Following the mindset of Lamont and Scott (1970) mass transfer at the bubble surface reflects the replacement of surface elements because of the local energy dissipationε in the surrounding liquid, originating from the CFD calculation and determined by the LES turbulence model.

(6) $$k_{L}=0.301\sqrt{\frac{\;D_{O_{2},L}}{\nu }}∙\sqrt[{4}]{\frac{\;\varepsilon }{\nu }}.$$

The solubility of oxygen $c_{O_{2}}^{*}$ is set by the Henry constant *H* and the volume fraction of oxygen in the air (y=0.21).

(7) $$c_{O_{2}}^{*}=\frac{y∙(P_{\mathrm{head}}+\rho_{L}\mathrm{hg})}{H}.$$

Therein, the local solubility considers the pressure in the headspace $P_{\mathrm{head}}$ in addition to the hydrostatic pressure expressed by the liquid height *h*, the liquid density $\rho_{L}$ and the gravity constant *g*. Notably, the hydrostatic pressure is also considered to calculate bubble sizes by applying the ideal gas law.

Fluid viscosity, density and surface tension were assumed to be similar to the properties of water. Walls are implemented as non‐slip surfaces. The top of the liquid is a free surface boundary where bubbles may exit the system. In accordance with the original process, a headspace overpressure of 0.55 bar was taken into account. Averaged simulated process data were saved every 0.01 s whereas local data for contour plots were recorded at 5 s intervals on four output planes. During the initiation of the simulation, the reactor system did not contain any pre‐existing bubbles. The emerging pseudo‐steady state predominantly relies on factors such as the reactor level, volume flow, and bubble size. As steady‐state indicator the average gas hold‐up and the total bubble count were tracked. Once these parameters are stabilized and maintained at a constant level, the evaluation of the simulation results were conducted, typically occurring after a minimum duration of 400 s. In EL simulations, bubbles are treated as point particles that do not displace water. Hence, the fill level was not affected by the bubble volume.

### 2.3 Measurement of *k*_*L*_ *a* Value

The determination of the experimental *k*_*L*_ *a* values was carried out in the reactor vessel using the degassing method. Instead of removing the dissolved oxygen by a nitrogen gas sweep, oxygen was removed using sodium sulfite and copper sulfate. A polarography Clark‐type electrode (manufactured by Yellow Springs Instrument Co.) was used to measure the oxygen activity. The electrode incorporated a Teflon membrane with a membrane thickness of 0.0254 mm. Four of these probes were placed in the fermenter space at different heights and axial positions. The tank has been filled to a volume of 470 m³ and adjusted to 32°C. Cupric sulfate was dissolved with water and the air flow rate was adjusted as indicated. The probes were set to zero, spanned, and the air turned off. Since the fermenter was air blown, the sodium sulfate was mixed in a separate tank and circulated through the fermenter until the sulfite was spent. The desired air flow rate was adjusted, and the DO curves were recorded. A series of air flow rates were tested. The calculations were made using Equation (8).

(8) $$k_{L}a=\frac{1}{t_{2}-t_{1}}∙\ln\left(\;\frac{c_{O_{2}}^{*}-c_{O_{2}}(t_{2})}{c_{O_{2}}^{*}-c_{O_{2}}(t_{1})}\right).$$

### 2.4 Mixing Time Evaluation

Mixing times of different operational conditions were determined via CFD simulations by applying pulse stimuli with glucose at the top right corner of the reactor. Glucose was injected into hydrostatic pseudo‐steady state flow fields. Glucose levels were monitored at three representative locations in the reactor. After glucose concentrations reached 95% of the theoretical maximum at each observation spot, the mixing time could be determined by calculating the average value. Based on the mixing time for a 90% dispersion, the mixing time for the 95% level can be estimated with Equation (9).

(9) $$\theta_{95}=\theta_{90}\frac{\ln(0.05)}{\ln(0.10)}.$$

The probes were positioned on the opposing side of the injection point at heights of 2, 7, and 12 m above the bottom. Each probe was located at a distance of 1 m from the reactor wall to ensure its placement inside of the heat exchange coil. Details can be found in Appendix B. The mixing time was solely assessed through CFD simulation and not through experimental observation.

### 2.5 One Compartment Model

Whereas computationally intensive CFD simulations are limited to snapshot analysis, entire fermentation runs may be well simulated by solving the differential equations (Equations (18), (19), (20), (21)), for example, using the Matlab framework. The simplifying approach intrinsically assumes an ideally mixed bioreactor, the so called one‐compartment model. Hence, likewise simulation results may be compared with the CFD predictions of the snapshot series. To ensure comparability, the same kinetic model is used (Equations (10), (11), (12), (13a), (13b), (14), (15), (16), (17)).

### 2.6 Kinetic Model

It is assumed that the uptake of substrate is constrained by the availability of oxygen and glucose according to the “limiting substrate theory” of Roels (1983). Although *E. coli* is able to metabolize glucose under anaerobic conditions, the production of l‐Phe is limited to oxygen availability. A graphical explanation of the cell model is provided in Appendix C. Hence, the glucose uptake model already reflects the application case of l‐Phe production. Thereon, the substrate uptake *q*_*S*_ comprises Monod‐type kinetics for oxygen and glucose limiting cell growth and product production. The total amount of substrate available for cells is determined in Equation (10) based on glucose and oxygen supply.

(10) $$q_{S}=q_{S,\max}∙\min\left(\frac{c_{S}}{c_{S}+k_{S}},\;\frac{c_{O_{2}}}{c_{O_{2}}+k_{O_{2}}}\right).$$

The cells utilize the substrate for growth (*q*_*S*_,_*growth*_), product formation (*q*_*S*_,_*prod*_), and maintenance $\left(m_{S}\right)$ needs:

(11) $$q_{S}=q_{S,\mathrm{growth}}+q_{S,\mathrm{prod}}+m_{S}=\frac{µ}{Y_{\mathrm{XS}}^{\mathrm{fit}}}+\frac{q_{P}}{Y_{\mathrm{PS}}^{\mathrm{fit}}}+m_{S}.$$

Notably, the kinetic model considers intrinsic preferences of the cells. The prior use of substrate is for maintenance. The remainder is assigned to product formation and growth ($q_{S,\mathrm{prod}+\mathrm{growth}}$) preferably channeling the substrate to l‐Phe production in the engineered strain:

(12) $$q_{S,\mathrm{prod}+\mathrm{growth}}=\max(\left(q_{S}-m_{S}\right),0).$$

Given that l‐Phe production (*q*_*p*_) is assumed to be partially growth coupled with a nongrowth‐associated contribution ($q_{P,\mathrm{non\_growth}})$

(13a) $$q_{P}=\frac{µ}{Y_{\mathrm{XP}}^{\mathrm{fit}}}+q_{P,\mathrm{non}\_\mathrm{growth}}$$

the related substrate consumption $q_{S,\mathrm{prod}}$ distinguishes between the growth‐coupled and nongrowth coupled terms:

(13b) $$q_{S,\mathrm{prod}}=q_{S,\mathrm{prod},\mathrm{growth}}+q_{S,\mathrm{prod},\mathrm{non}\_\mathrm{growth}}.$$

The model considers that substrate is first consumed for the nongrowth coupled product formation $\frac{m_{P}}{Y_{\mathrm{PS}}^{\mathrm{fit}}}$

(14) $$q_{S,\mathrm{growth}}+q_{S,\mathrm{prod},\mathrm{growth}}=\max\left(\left(q_{S,\mathrm{prod}+\mathrm{growth}}-\frac{m_{P}}{Y_{\mathrm{PS}}^{\mathrm{fit}}}\right),0\right)$$

before using the last fraction for growth coupled product formation $q_{S,\mathrm{prod},\mathrm{growth}}$ and biomass formation.

The parameter $m_{P}$ denotes the maximum non growth related product synthesis rate.

(15) $$q_{P,\mathrm{non}\_\mathrm{growth}}=\left\{\begin{array}{l} m_{P}\;\mathrm{if}\;q_{S,\mathrm{prod}+\mathrm{growth}}>m_{P}/Y_{\mathrm{PS}}^{\mathrm{fit}},\\ q_{S,\mathrm{prod}+\mathrm{growth}}\cdot Y_{\mathrm{PS}}^{\mathrm{fit}}\;\mathrm{if}\;q_{S,\mathrm{prod}+\mathrm{growth}}<m_{P}/Y_{\mathrm{PS}}^{\mathrm{fit}}. \end{array}\right.$$

The growth rate µ is obtained from Equation (16) and considers only the substrate fraction used for growth ($q_{S,\mathrm{growth}}$) and growth related product synthesis $q_{S,\mathrm{growth}}+q_{S,\mathrm{prod},\mathrm{growth}}$.

(16) $$µ=\frac{q_{S,\mathrm{growth}}+q_{S,\mathrm{prod},\mathrm{growth}}}{\frac{1}{Y_{\mathrm{XS}}^{\mathrm{fit}}}+\frac{1}{Y_{\mathrm{PS}}^{\mathrm{fit}}\cdot Y_{\mathrm{XP}}^{\mathrm{fit}}}}.$$

The oxygen uptake rate is growth‐coupled by assuming that the biomass per oxygen yield remains constant:

(17) $$q_{O_{2}}=\frac{µ}{Y_{{\text{XO}}_{2}}}.$$

Process models considering mass balances for the components biomass (X), substrate (S), product (P) and dissolved oxygen (O_2_) lead to the differential Equations (18)–(21).

(18) $${\dot{c}}_{X}=\left(µ-\frac{F_{\mathrm{in}}^{\mathrm{liq}}}{V}\right)c_{X}$$

(19) $${\dot{c}}_{S}=q_{S}c_{X}-\frac{(c_{S,F}-c_{S})F_{\mathrm{in}}^{\mathrm{liq}}}{V}$$

(20) $${\dot{c}}_{P}=q_{P}c_{X}-\frac{c_{P}F_{\mathrm{in}}^{\mathrm{liq}}}{V}$$

(21) $${\dot{c}}_{O_{2}}=k_{L}a(c_{O_{2}}^{*}-c_{O_{2}})-q_{O_{2}}c_{X}$$

The rates $q_{S}$, $q_{P}$, and $q_{O_{2}}$ are given in Equations (10), (13a), and (17). The parameters $k_{S}$, $k_{O_{2}},Y_{{\mathrm{XO}}_{2}}$ and ${µ}_{\max}$ were taken for *E. coli* from Xu, Jahic, and Enfors (1999), Morchain Gabelle, and Cockx (2013). The maximal substrate uptake rate $q_{S,\max}$ was determined by Equation (11), where *µ* was replaced with ${µ}_{\max}$. The model parameters $Y_{\mathrm{XS}}^{\mathrm{fit}}$,$\;Y_{\mathrm{PS}}^{\mathrm{fit}}$,$\;Y_{\mathrm{XP}}^{\mathrm{fit}}$, and $m_{P}$ were fitted by minimizing the sum of least‐squares between the simulated and the measured process data using the Matlab function *lsqcurvefit()*. The results obtained from the three different datasets were averaged. Hence, the kinetic model represents a hybrid approach of independently identified kinetic parameters of lab tests and large‐scale observations analyzing the simplifying one‐compartment approach. Anticipating that large scale inhomogeneities particularly bias the cellular capacity for draining carbon into biomass and product (Löffler et al. 2016) the yield coefficients, that are derived from real large scale data, intrinsically reflect real industrial stress. Accordingly, the fitted parameters differ from results that would have been obtained in independent small‐scale experiments with the strain. Table 1 comprises all kinetic parameters.

**Table 1:** Overview of the parameters of the kinetic model and their source. The errors are the standard deviations obtained from the three data sets.

Parameter	Value	Source
${µ}_{\max}$	0.6 h^−1^	Xu, Jahic, and Enfors (1999); Morchain, Gabelle, and Cockx (2013)
$q_{S,\max}$	3.96 g g^−1^ h^−1^	Equation (11) by *µ* = *µ*_max_
$k_{S}$	0.05 g L^−1^	Xu, Jahic, and Enfors (1999); Morchain, Gabelle, and Cockx (2013)
$k_{O_{2}}$	3.125 ∙ 10^−3 ^mmol L^−1^	Xu, Jahic, and Enfors (1999); Morchain, Gabelle, and Cockx (2013)
$Y_{{\mathrm{XO}}_{2}}$	0.033 mmol g^−1^	Xu, Jahic, and Enfors (1999); Morchain, Gabelle, and Cockx (2013)
$m_{S}$	0.04 h^−1^	Morchain, Gabelle, and Cockx (2013); Xu, Jahic, and Enfors (1999)
$Y_{\mathrm{XS}}^{\mathrm{fit}}$	0.18 ± 0.01 g g^−1^	Fitted
$Y_{\mathrm{PS}}^{\mathrm{fit}}$	0.34 ± 0.01 g g^−1^	Fitted
$Y_{\mathrm{XP}}^{\mathrm{fit}}$	19.03 ± 1.19 g g^−1^	Fitted
$m_{P}$	0.14 ± 0.01 h^−1^	Fitted

The biomass concentration was assumed to be uniformly distributed and constant within the time window of CFD simulation (several hundred seconds). For the CFD simulations, five time points of real l‐Phe production were chosen. Operating conditions such as filling level, biomass concentration, air volume flow, and glucose feed rate were adjusted accordingly (Table 2).

**Table 2:** Overview of the operating parameters used in the CFD simulations of five different fermentation times.

Fermentation times	Cultivation time (h^−1^)	Biomass (g L^−1^]	Air flow (m³ min^−1^)	Glucose feed (kg min^−1)^	Reactor liquid volume (m^3^)
1	9	9.48	344	64	396
2	12	12.09	352	88	410
3	16	16.89	341	94	433
4	20	19.32	344	92	455
5	24	18.72	349	89	475

## 3 Results and Discussion

### 3.1 Bubble Size Distribution and Mass Transfer

Upon attaining a pseudo‐equilibrium state, the bubble column contained up to 18 billion bubbles when the reactor reached its full capacity with the air flow of 1 vvm. This was primarily due to high bubble breakage rates in the strongly turbulent region close to the air inlet which dominate the local BSD. According to the breakage model by (Mast and Takors 2023a) turbulent dissipation energy in the bubble environment is the decisive factor for bubble break‐up. Consequently, the starting diameter of 10 mm leads to a BSD with 2.30 mm of arithmetic bubble diameter and 3.03 mm of Sauter diameter *d*_*32*_ for 470 m^3^ filling volume operating at 1 vvm.

Given that the bubble diameter *d* determines drag forces which in turn heavily influence bubble‐liquid dynamics (Gradov et al. 2017) the mixing conditions inside the bioreactor may be best understood by analyzing *d*. The diameter plays a pivotal role for determining the bubble rise velocity, gas hold‐up, mass transfer, and for describing the impact of bubbles on the fluid motion. Turbulent dispersion forces and swarm effects were not accounted for in this study, potentially impacting the results (McClure et al. 2015). Addressing bubble swarm under similar conditions would necessitate evaluating the environment for each bubble at every time step, making the simulation computationally even more intensive finally extending the scope of this study. Solutions to overcome this challenge are needed. Nonetheless, the motion of bubbles here is primarily influenced by the fast‐circulating fluid, minimizing the impact of turbulent dispersion forces and swarm effects compared to other reactor designs. In the given setup the bubble column attained a maximum gas hold‐up of 23.31% and liquid velocities up to 4.4 m s^−1^ near the air injection points. Even slight modifications of the energy input or gas flow may strongly affect the bubble breakage rate and thus change the liquid flow field. Rising bubbles not only cause local eddies but also large fluid movement by pushing the fluid upwards before recirculating down through the channel between the wall and the heat exchange coil. The difference of gas hold‐up between the riser and the downcomer serves as the driving force for the liquid circulation in the airlift reactor (Wadaugsorn et al. 2016). A fraction of bubbles was pushed sidewards before following the downward fluid movement leading to an accumulation of small bubbles at the bottom of the reactor. Most bubbles, however, left the system a few seconds after injection on a direct path toward the liquid surface. Peak ascending velocities reached 4.11 m s^−1^. The bubble population averages with 0.50 m s^−1^. Notably, these absolute velocities should be relativized considering the correlated slip velocities 1.97 and 0.20 m s^−1^, respectively. The difference mimics the strong fluid motions inside the bioreactor.

**Figure 2:**
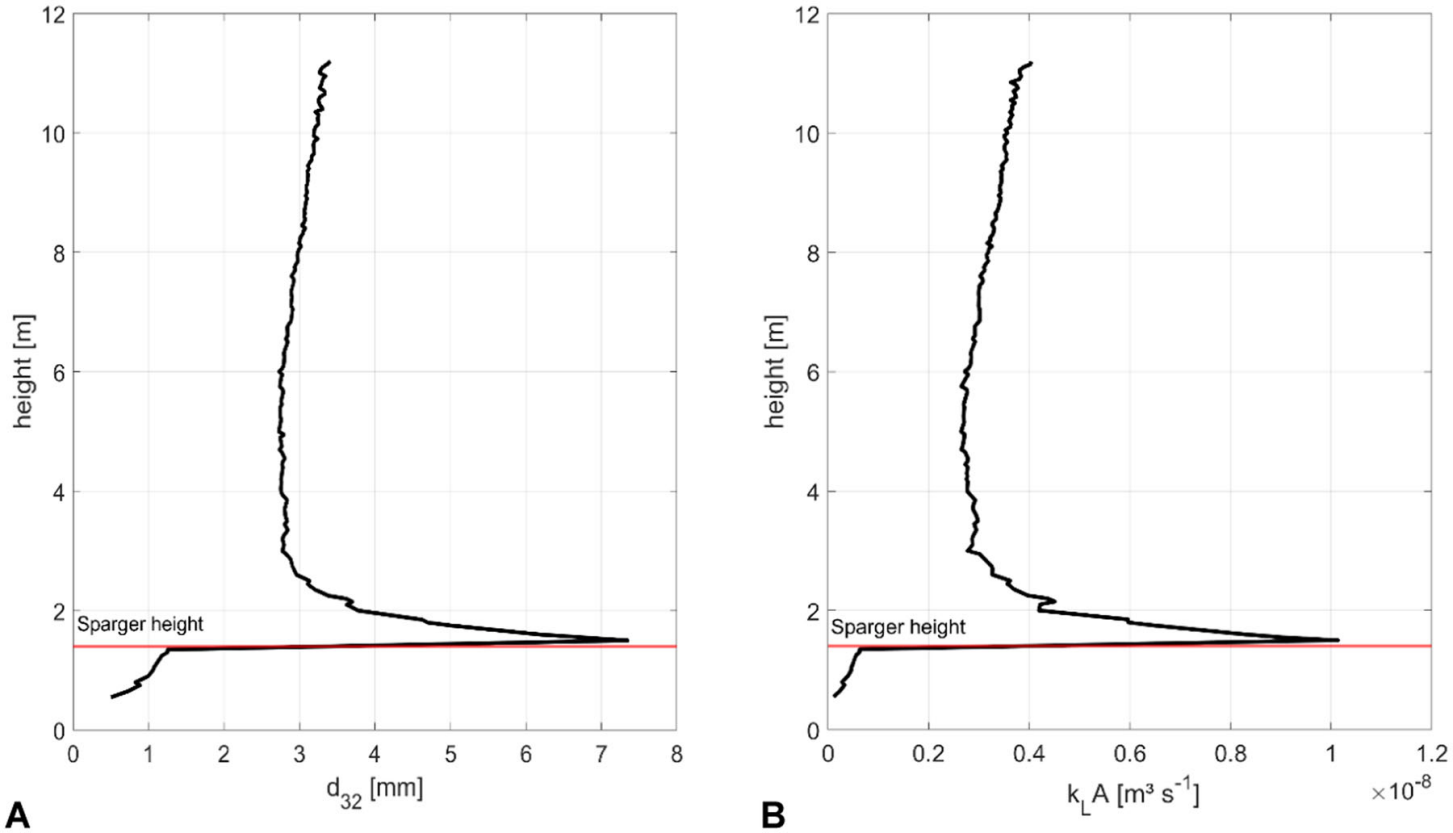
Dependence of the reactor height on (A) the Sauter diameter d_32_ and (B) the *k_L_A* value. In each case, an average value of all bubbles at the same height was calculated.

**Figure D1:**
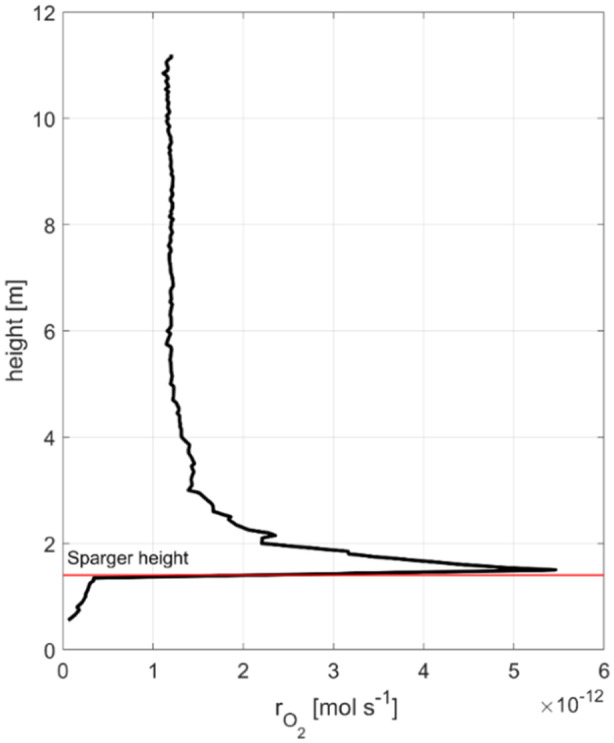
Dependence of the oxygen mass transfer rate on the reactor height. An average value of all bubbles at the same height was calculated. It was assumed that the fluid was 20% saturated with oxygen.

In Figure 2A the local Sauter diameter varies significantly with the axial height. Below the sparger (1.4 m) bubble sizes range between 0.5 and 1.3 mm. Recirculating bubbles from the downcomer experienced prolonged residence times. They encountered more frequent and intensified turbulent eddies which subsequently lead to the fragmentation into smaller bubbles. Due to their reduced size, the buoyancy force is inadequate to counteract gravitational forces resulting in the temporary entrapment of these bubbles at the bottom of the reactor. Because of the injection of new bubbles at the sparger there is an abrupt increase of *d*_32_ to 7.4 mm. However, the model predicts that these bubbles undergo immediate breakage because of the locally high energy dissipation rates. Above the sparger the smallest Sauter diameter of 3.0 mm was found at a height of 2.77 m. Then, *d*_32_ increases continuously with height reaching a maximum value of 3.36 mm at the liquid surface (11.55 m). This mimics the reduction of the hydrodynamic pressure. Figure 2B illustrates the height dependent profile of *k*_*L*_ *A* which shows a similar pattern to *d*_*32*_. Noteworthy, *k*_*L*_ *A* is computed by multiplying the mass transfer coefficient *k*_*L*_ (Equation 4), with the integral surface area of all bubbles at the corresponding height. The maximum value of 1.01 × 10^−8^ m^3^ s^−1^ is achieved directly above the sparger. Then, values reduce to 0.27 × 10^−8^ m^3^ s^−1^ at 3 m before they rise toward the surface. Both, the highest *k*_*L*_ *A* values at the sparger as well as the rising trend upwards mirror the proportionality to *d*_32_^1.5^ which is characteristic not only for the film‐theory (Equation 4) but also, for the penetration theory (Equation 5). The correlations reveal a substantial impact of *d*_32_ on mass transfer, highlighting its spatial variability as a critical determinant for the emergence of oxygen gradients within the reactor. As shown in Figure D1 in the Appendix D, the influence of hydrodynamic pressure on the maximum oxygen solubility introduced an additional dependency on the reactor height. Consequently, the mass transfer is highest at the sparger and remains stable between 3 and 6 m height. With increasing height, the maximum solubility of oxygen decreases leading to reduced concentration gradients as a driving force. On the other hand, the rising bubbles experience decreasing hydrostatic pressure, leading to expansion and the creation of a larger phase exchange area, thereby enhancing mass transport. The superposition of both phenomena led to a complex oxygen transfer scenario which is nonlinear to the height of the bubble column.

**Figure 3:**
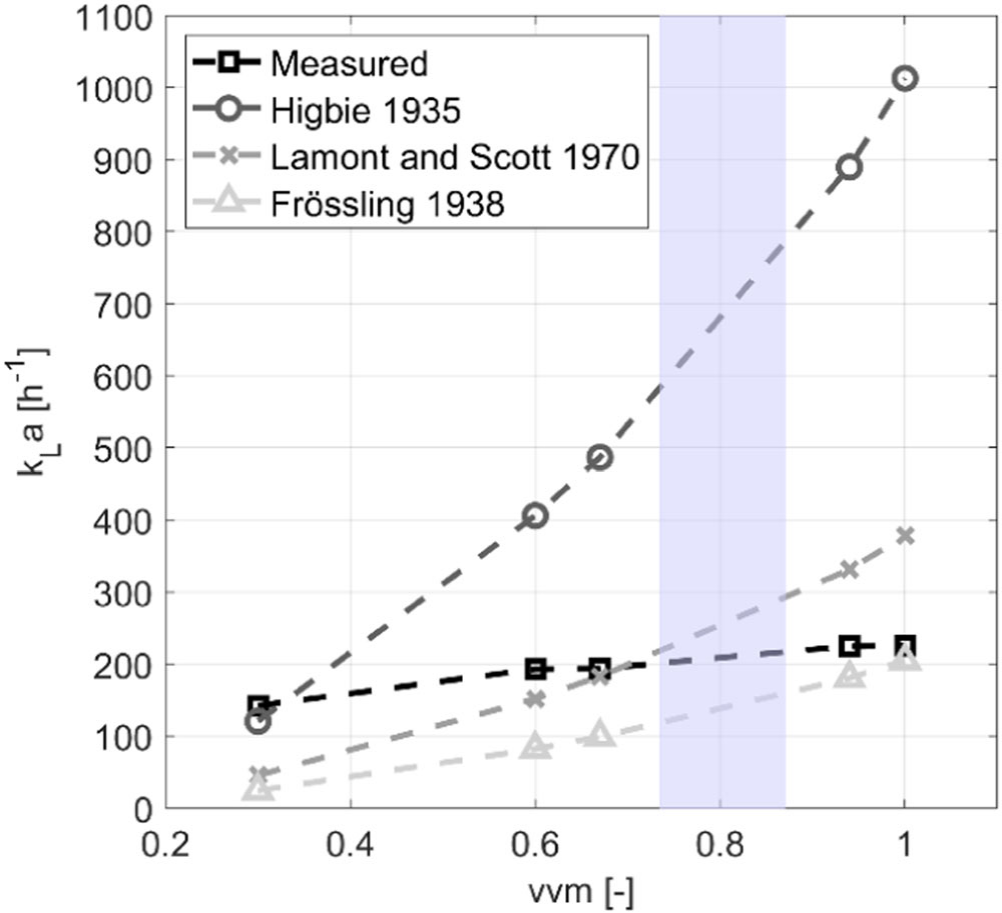
The k_L_a values, determined via the models by Higbie, Frossling, and Lamont and Scott for different air volume flow rates, are compared with experimentally determined values. The shaded area represents the operational range of the fermentation.

Figure 3 illustrates the impact of the aeration rate on the *k*_*L*_ *a* value, calculated with the models of Higbie (1935), Lamont and Scott (1970), Frossling (1938), and compared to measured data. Experiments with 470 m^3^ filling volume found the lowest *k*_*L*_ *a* of 142 h^−1^ for vvm of 0.3 and the maximum of 226 h^−1^ for 1 vvm. The rise of *k*_*L*_ *a* was expected, given the concurrent increase in gas hold‐up, as demonstrated in Appendix E. Consequently, this leads to an expansion of the gas hold‐up and the mass transfer surface. Noteworthy, estimated *k*_*L*_ values diverge considerably with respect to the different models. The Lamont and Scott (1970) approach underestimated *k*_*L*_ *a* by 97 s⁻¹ at low vvm of 0.3 but overestimated strongly by 153 s⁻¹ at 1 vvm. Instead of estimating the replacement of surface elements via contact time, Lamont and Scott (1970) estimate the replacement of surface elements through the turbulent eddy dissipation rate in the bubble environment. Whereas this assumption is fulfilled for wall friction and close to impellers (Alves, Vasconcelos, and Orvalho 2006) the condition seems to be not valid for the current scenario. Here, ε is not strictly correlated with vvm.

Surprising enough, the well‐known (Higbie 1935) approach overestimated measured *k*_*L*_ *a* values strongly with rising vvm. Higbie's model was found explicitly suitable for non‐stirred reactors where the slip velocity plays a crucial role. The observed disparity with the measurement in the current case may hint to an intrinsic misfit. One option is the potential overestimation of *a* via gas hold‐up which reaches 23.3% at 1 vvm (see Appendix E). Although in the reasonable range for bubble columns, too high *a* values cannot be excluded as the local maximum energy dissipation of 133 W m^−3^ exceeds the investigated bubble breakage range by (Mast and Takors 2023a). Unfortunately, no experimental data were available for ε, *a*, and *d*_*32*_. Frankly speaking, this may mirror a typical condition of industrial routine in production plants when explicit effort is put on the proper operation of the reactors rather than their detailed physical characterization. Consequently, it was not possible to conclusively determine which factor was responsible for the overestimation of the *k*_*L*_ *a* value. However, these values are strongly interdependent. Hence, propagating errors immediately influence each other. Furthermore, impacts of bubble coalescence reducing *a* were not considered yet. According to (Buchholz et al. 1978) strong aeration conditions are prone for bubble coalescence. On contrary, the current media composition comprising multiple salts should prevent pronounced coalescence (Craig, Ninham, and Pashley 1993). In general, predicting bubble coalescence remains challenging. Either well‐validated models for EL frameworks are lacking or approaches are too computationally intensive because local criteria must repeatedly be checked (Boshenyatov 2012). Measuring *k*_*L*_ *a* in large volumes is complex and prone to errors. While the sodium sulfite and copper sulfate method avoids the need for extensive nitrogen usage in comparison to the nitrogen sweep method, it may still introduce inaccuracies due to delayed reactions and the variable effects of the copper catalyst. These factors could explain discrepancies between experimental findings and theoretical predictions as well.

The Frossling (1938) model consistently underestimated measured *k*_*L*_ *a* values in particular for the lowest vvm. However, simulations converge to experimental data with higher vvm finally even agreeing for 1 vvm. Interestingly Eibl et al. (2020) recommended to employ the Frossling (1938) model for bubble diameters smaller than 3 mm. This holds true for the current scenario. Considering the operational range from 0.73 to 0.87 vvm (Figure 3), the Frossling model performed best which is why it was chosen for the following simulations.

### 3.2 Mixing Characteristics

**Figure 4:**
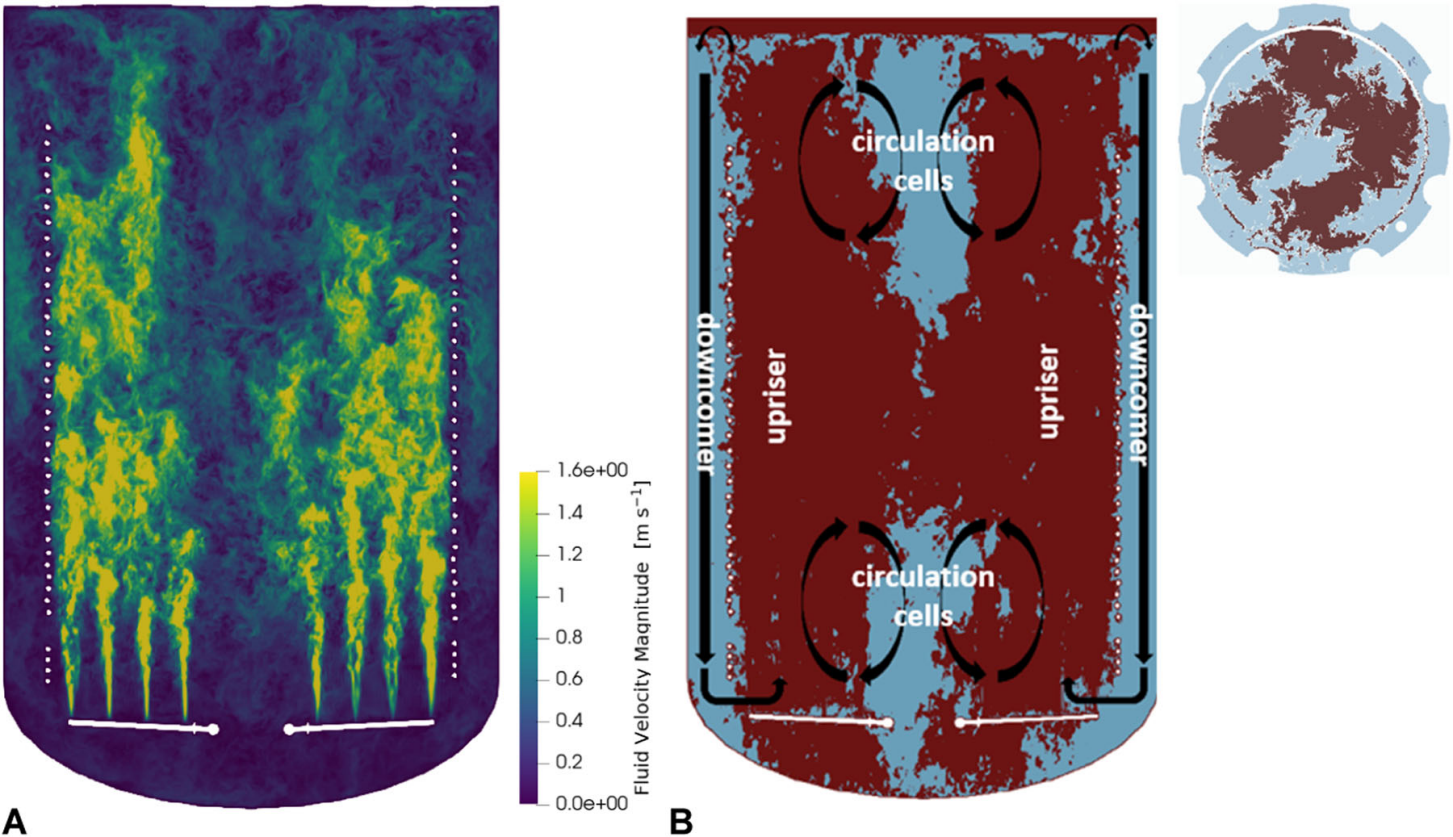
Velocity fields on a plane intersecting the reactor's central vertical plane. The reactor is filled entirely and operated at 1 vvm. (A) Velocity magnitude field. (B) Regions of ascending fluid (red) and descending fluid (light blue). Additionally, a horizontal plane intersecting the reactor horizontal at a height of 5.5 m is displayed.

Figure 4 depicts a contour plot of the fluid velocity magnitude on a vertical plane intersecting the reactor.

The reactor was completely filled. Air is injected with 1 vvm which induces the highest fluid velocities at the spargers that are mounted aside the middle of the bioreactor (Figure 4A). Above the center, most of the vortices dissipate and complex reflux vortices are created finally causing vertical velocity gradients between the center and the surroundings. Below the spargers, hardly any fluid movement was observable. Only moderate velocities of maximum 1 m s^−1^ were found between the cooling coils and the reactor wall, the so‐called ‘downcomer’. Apparently, low shear stress dominates this zone which returns the fluids narrowly above the sparger. Figure 4B provides a snapshot illustrating the upward (in red) and downward (in light blue) fluid flow with the black arrows indicating the direction of the liquid movement. As anticipated by the relatively low fluid velocity in the downcomer only a fraction of the upwards transported fluid was transported back via this route. The rest flowed back through the center of the bioreactor creating large vortices. The discussed flow profile is further corroborated by the velocity vector field presented in Appendix F.

Notably, multiple similar vortices existed on different levels creating fluid movements left and right of the centerline. As a consequence, a fraction of the fluid remained circulating within one reactor half. The fluid pattern mirrors the missing impact of uprising bubbles in the center of the bioreactor. Such phenomena has been already described by (Millies and Mewes 1995) as “circulation cells” with the particular characteristic that fluid elements remain entrapped in this vortex.

Summarizing Figure 4 illustrates that the flow pattern in the bubble column may be best described by introducing three zones: (1) the upriser, driven by the uprising bubbles, located above the sparger and restricted by the cooling coils, (2) the downcomer, located between the reactor wall and the cooling coils and (3) the circulation cells, rotating next to the centerline radially ranging to the upriser. The impact of rising bubble is of crucial importance to establish these zones.

**Figure 5:**
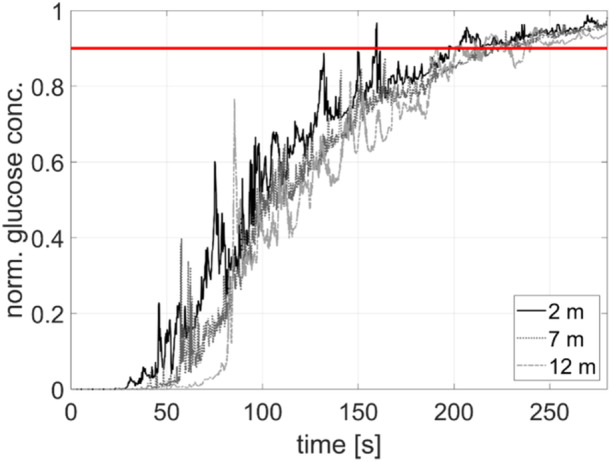
Normalized glucose concentration over time at sensors located at heights of 2, 7, and 12 m from bottom after glucose injection at time = 0. Glucose was added 1 m below the liquid surface, one‐sided on the opposite side of the sensors (details given in Figure B1). The reactor was fully filled and operated at a flow rate of 1 vvm. The red line marks the 90% mixing criterium.

**Figure B1:**
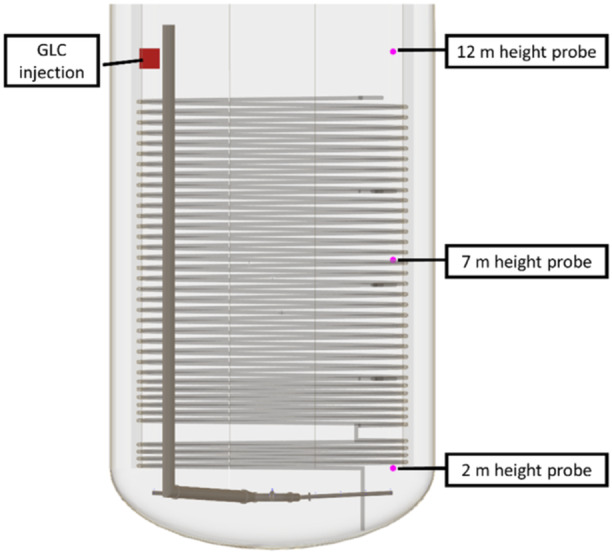
Reactor configuration featuring highlighted positions of three probes at heights of 2, 7, and 12 m for the mixing experiments. The red square on the right indicates the initial glucose injection point.

Figure 5 shows the temporal evolution of the normalized glucose concentration at three sensor locations (2, 7, and 12 m from bottom) after the addition of the glucose tracer, as shown in Figure B1 (Appendix B).

Mixing time simulations revealed that the tracer remained undetected by the sensors for 27 *s* which defines the convective transport time from the injection spot to the other side of the reactor. The linked flows between upriser and downcomer are reflected by the periodic fluctuations at the probes compared to the rather steady increase of the mean concentration. Given the noisy peak patterns a valid estimation of cycling time, indicated by the time difference of subsequent peaks, is hardly possible (Figure 5). As the probes (at 2, 7, and 12 m) detected mixing efficiency of 90% after 202.9, 224.2, and 240.3 s, respectively, the average mixing time corresponds to 222.5 s which equals 289.4 *s* on 95% confidence level. Compared to the mixing time of around 140 s estimated for another bubble column (490 m^3^ operated with 0.58 vvm(Bisgaard et al. 2022) current mixing is slower. Apparently, the long mixing times of the current column are the consequence of the rather compact geometry (H/D of 1.6 compared to 4.9 in (Bisgaard et al. 2022) which also causes the installation of ‘circulation cells’ that entrap liquid elements.

**Figure 6:**
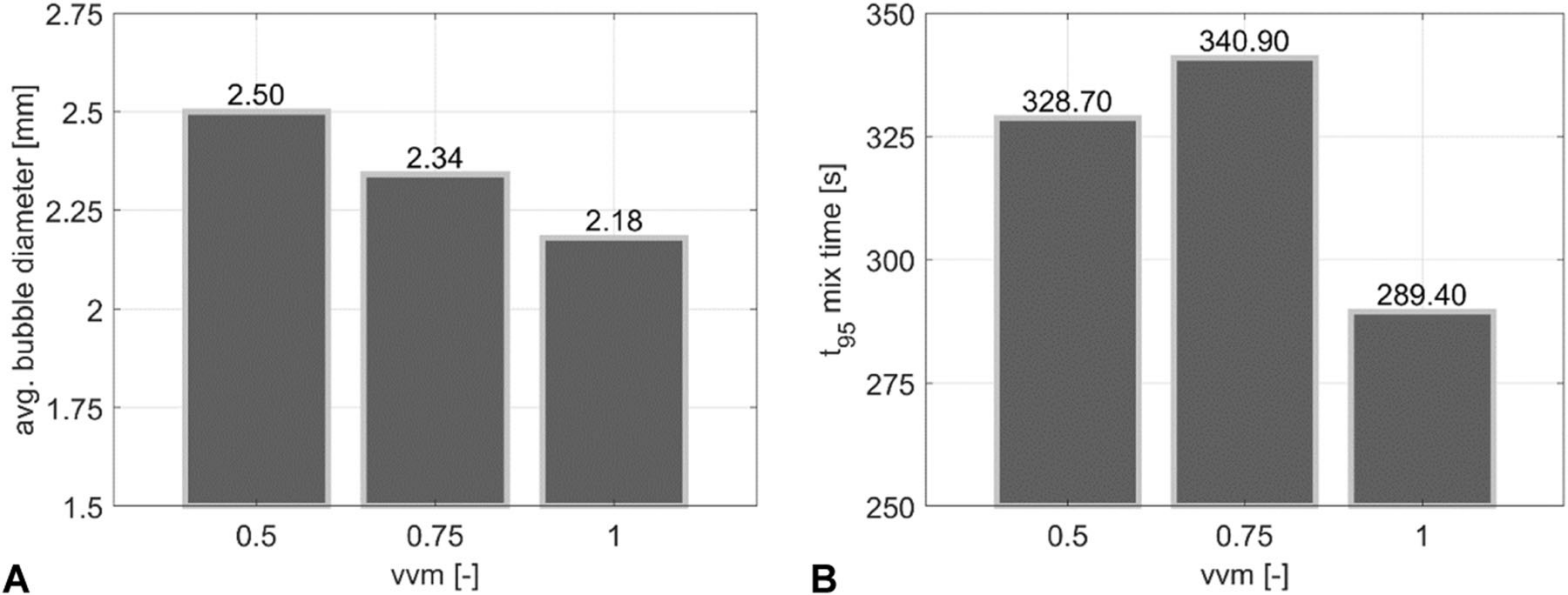
Impact of varying air injection volumes on (A) the average bubble diameter and (B) the mixing time (95% confidence level).

The impact of air flow rate on hydrodynamics is presented in Figure 6A,B.

With rising aeration rates from 0.5 to 1 vvm mean bubble diameters decreased from 2.5 to 2.18 mm, respectively. The trend mirrors the elevated energy input resulting in increasing gas hold‐up, liquid velocities, and turbulent dissipation energy, all in accordance with experimental observations (Akita and Yoshida 1974; Couvert, Roustan, and Chatellier 1999; Wongsuchoto 2003; Wild, Mast, and Takors 2023). Exalted local energy dissipation leads to increased bubble breakage (Mast and Takors 2023a) which coincides with broadened bubble size distributions (Siebler et al. 2019).

Typically, increased air flow should lead to reduced mixing times (Lu, Hwang, and Chang 1994; Gavrilescu and Tudose 1997; Wadaugsorn et al. 2016). Indeed, Figure 6B depicts the shortest mixing time of 289.4 s at 1 vvm. However, there is no linear increase to the value of 328.7 s at 0.5 vvm. Instead, mixing time peaks at 0.75 vvm with 340.9 *s*. The finding underpins the complex interaction of the three dominating flow zones outlining those intensities of upriser, downcomer, and circulating cells must be well equilibrated to optimize mixing.

**Figure 7:**
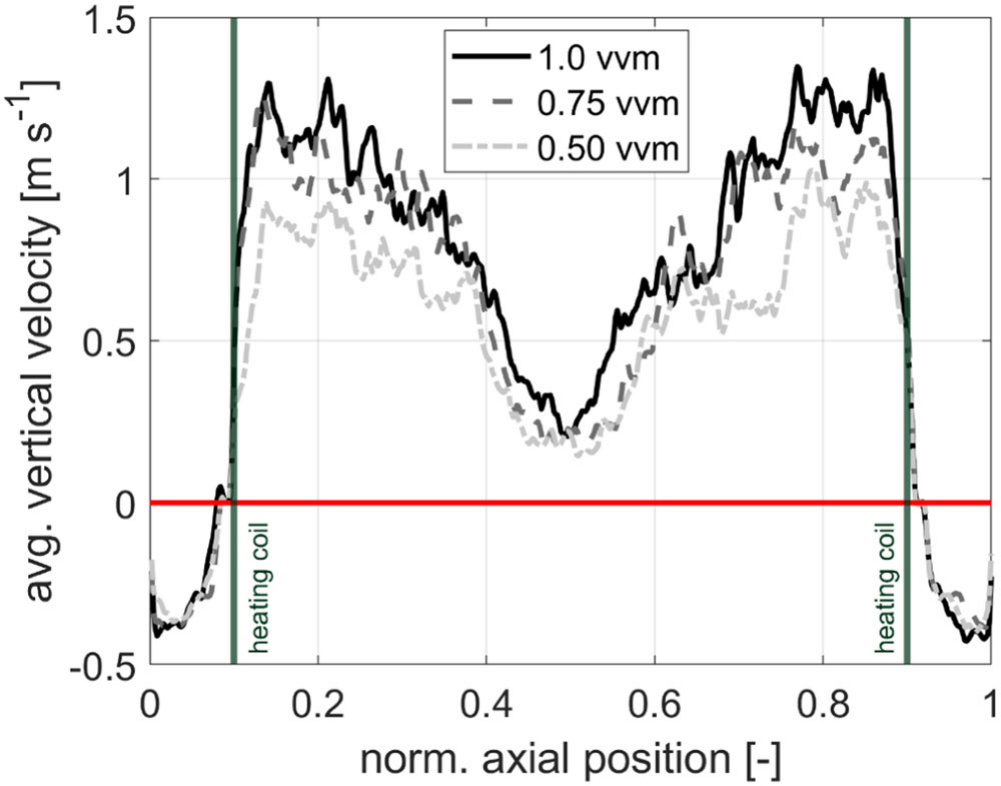
The vertical fluid velocity along a horizontal line intersecting the reactor at its midpoint and positioned at 5.5 m height.

Notably, the capacity of the downcomer is restricted as illustrated in Figure 7. Increasing vvm caused elevated ascending liquid flows in the upriser. On contrary, fluid flows in the downcomer are limited as (i) wall friction effects oppose rising velocities and (2) the fluid behaves almost incompressible due to the relatively low gas content. Consequently, the intensity of the circulating cells rises thereby increasing mixing times at 0.75 vvm. Further increased air flow to 1 vvm injected enough energy to create chaotic flow patterns affecting the circulations cells. As a result, mixing performance improves again.

### 3.3 Modeling Large‐Scale Production Performance

For modeling large‐scale performance microbial kinetics should be embedded in CFD simulations (Schmalzriedt et al. 2003). As a prerequisite, the missing parameters $Y_{\mathrm{XS}}^{\mathrm{fit}}$, $Y_{\mathrm{PS}}^{\mathrm{fit}}$, $Y_{\mathrm{XP}}^{\mathrm{fit}}$, $m_{P}\;$need to be estimated. Anticipating that large‐scale inhomogeneities particularly effect the cellular capacity for draining carbon into biomass and product (Löffler et al. 2016) said parameters were identified by nonlinear regression from real large‐scale measurements treating the bioreactor as a single compartment. Hence, impacts of large‐scale stress are intrinsically considered in the identified yield coefficients. As such, the model identification approach is in line with independent studies (Bisgaard et al. 2022) aiming to derive model‐based predictions for large‐scale.

**Figure 8:**
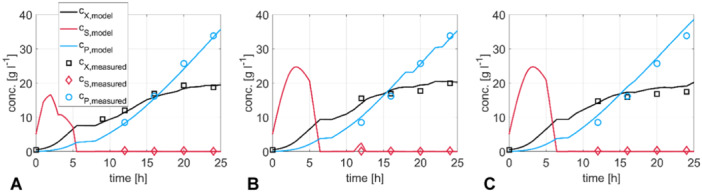
Concentration curves of substrates, biomass, and product over time. The solid line represents the results of the one compartment model, and the dots correspond to measured values. (A) Run 1, (B) Run 2, and (C) Run 3.

Figure 8 depicts that the combined fed‐batch and kinetic model is well able to simulate process conditions even considering different air and feed flows of the three runs. Details about glucose feed rates and volume air injection rates are given in Appendix G. The results show that the kinetic model could be well fitted to the experimental values. The observation that cell growth is hampered after 15 h is correctly reflected by the microbial model which prefers nongrowth‐related product synthesis to cell growth in case of substrate limitation. The low $Y_{\mathrm{XS}}^{\mathrm{fit}}$ of 0.18 g g^−1^ reflects the large carbon drain into product synthesis. Accumulated substrate is depleted within 6 h remaining in low mg L^−1^ levels as confirmed by measurements. Product formation is dominated by growth‐independent production as indicated by the linear rise of product concentrations and mirrored by the high *m*_*P*_ value of 0.14 h^−1^.

**Figure 9:**
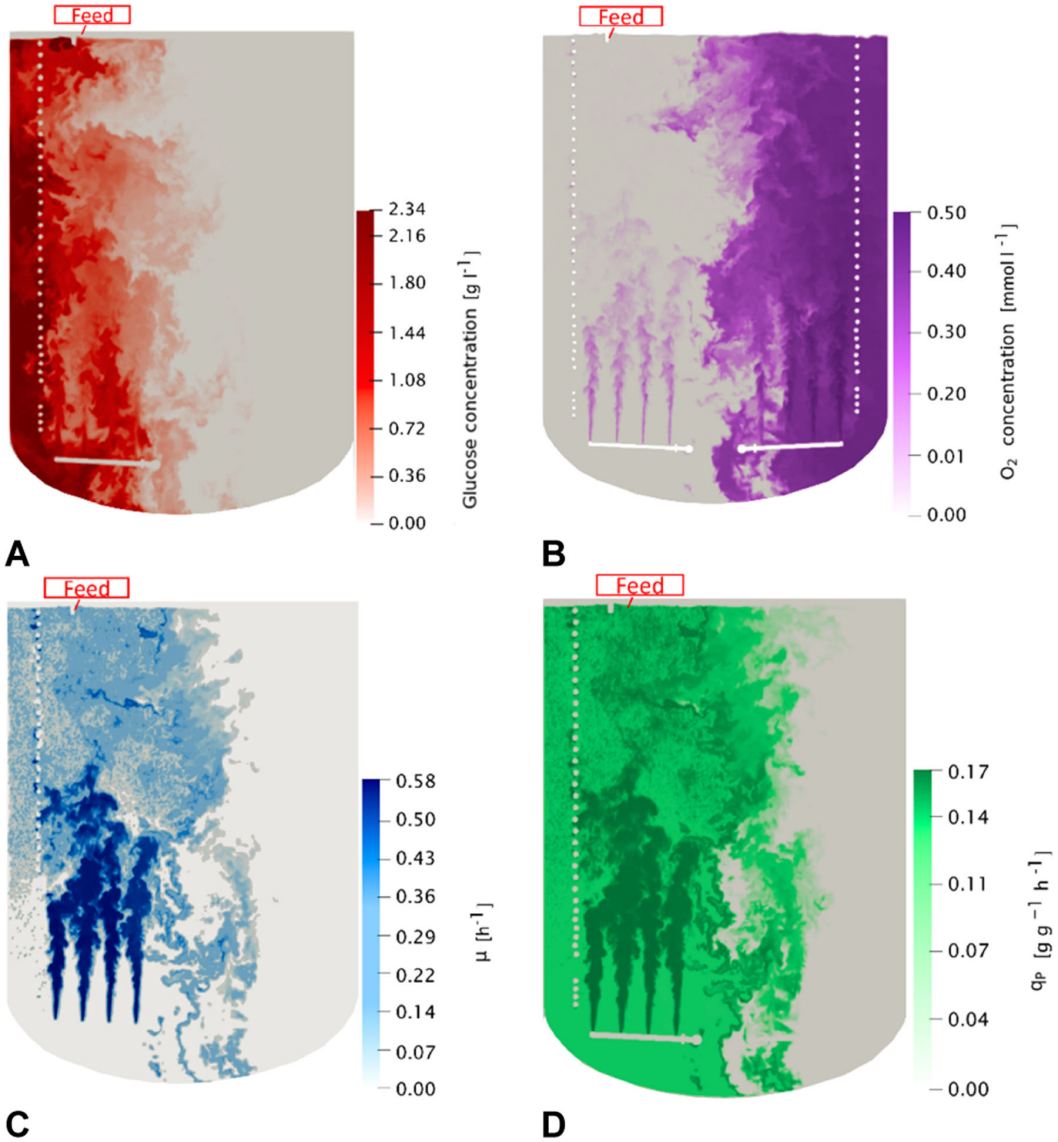
Contour plots on a plane intersecting the reactor's central vertical plane. The CFD simulation was calculated with process parameters according to fermentation time 4 with the maximum biomass concentration of 19.32 g L^−1^ after a fermentation of 20 h. Position of feeding inlet is shown. (A) Glucose concentration, (B) oxygen concentration, (C) growth rate, and (D) specific product synthesis rate.

The embedment of microbial kinetics in the CFD simulations lead to the contour plots for the concentrations of glucose, dissolved oxygen, growth and specific product formation rates as illustrated in Figure 9. The snapshot exemplifies conditions on a vertical plane bisecting the reactor. Fermentation time 4 of Table 2 with the maximum biomass concentration of 19 g L^−1^ is shown considering the real scenario of a single feeding spot at the top left.

Obviously, operational conditions and performance are split by the centerline. On the left side, high glucose and low oxygen levels were found, vice versa on the right. Microbial growth is predominately limited to the proximity of air inlet, and with few exceptions located above and between the spargers (Figure 9C). Given the fast glucose consumption rates compared to the long mixing times glucose is predominately consumed close to the injection spot co‐consuming oxygen. Hence, strong glucose‐limitation in other bioreactor zones lead to increased dissolved oxygen levels as the metabolic activity is slowed down. By comparison, the zone of product formation is extended covering more than half of the left wing of the bioreactor (Figure 9D). Both, cellular growth and product formation mirror key features of the microbial kinetics, namely (i) the preferred use of glucose for product formation, (ii) the crucial need of oxygen for growth and product formation, and (iii) the significant fraction of growth‐decoupled product formation. In other words, the availability of oxygen limits glucose uptake on the left side which in turn limits growth and, somewhat minor, production formation. Residual sugar remained on the left side which finally led to the bisectional distribution of sugar and dissolved oxygen. The latter is rather sporadically transported through the central region of the bioreactor. Thus, oxygen transfer predominately happened in close proximity to the bubbles that remain on one side. In essence, Figure 9 illustrates that long mixing times exceed the microbial need for refilling local nutrient supply. Consequently, the real space‐time yield of the bioreactor is below its optimum as only half of the volume is exploited.

**Figure 10:**
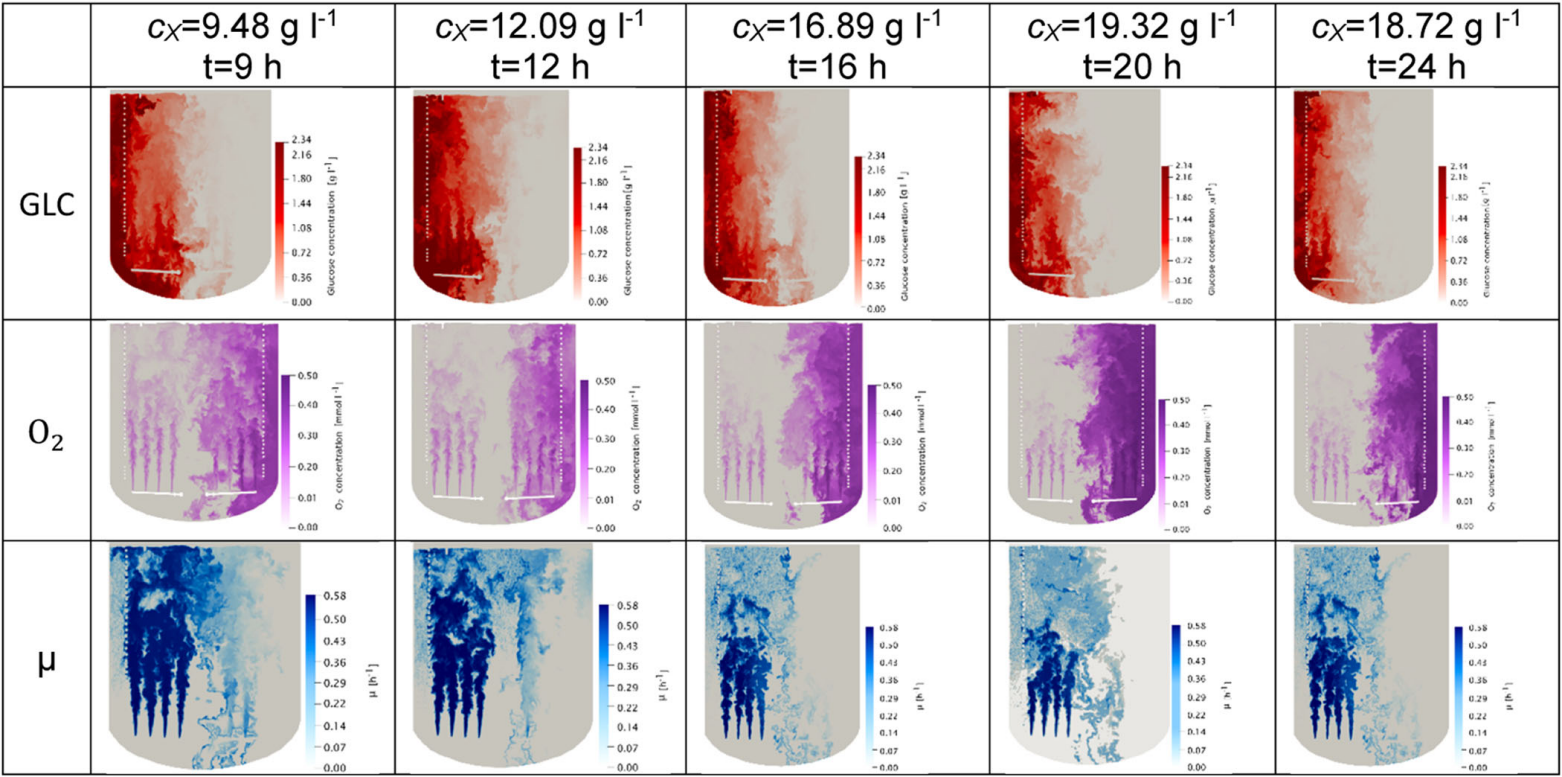
Contour plots of glucose, oxygen and growth rate on a plane intersecting the reactor's central vertical plane. Each contour shown for the five fermentation times after 9, 12, 16, 20 and 24 h.

Interestingly enough, the basic observation holds true for all investigated fermentation times of Figure 10. Although improved growth conditions were found for the lowest biomass concentration of 9 g L^−1^, the growth zone is still restricted. Highest glucose levels were observed at 12 h which coincide with high feed rate of up to 88 kg min^−1^ (Table 2). The kinetic cell model is designed with a major correlation between oxygen consumption and cell growth, reflecting reduced oxygen demand during the prioritized product synthesis. Despite peak biomass concentration occurring at 20 h, biomass growth is comparatively lower at this time point. As a result, oxygen consumption decreases, resulting in higher oxygen concentrations in the broth (Figure 10). Nevertheless, all cases show the already described strong gradients that may cause notable heterogeneities in the biological population (Morchain, Gabelle, and Cockx 2014).

**Figure 11:**
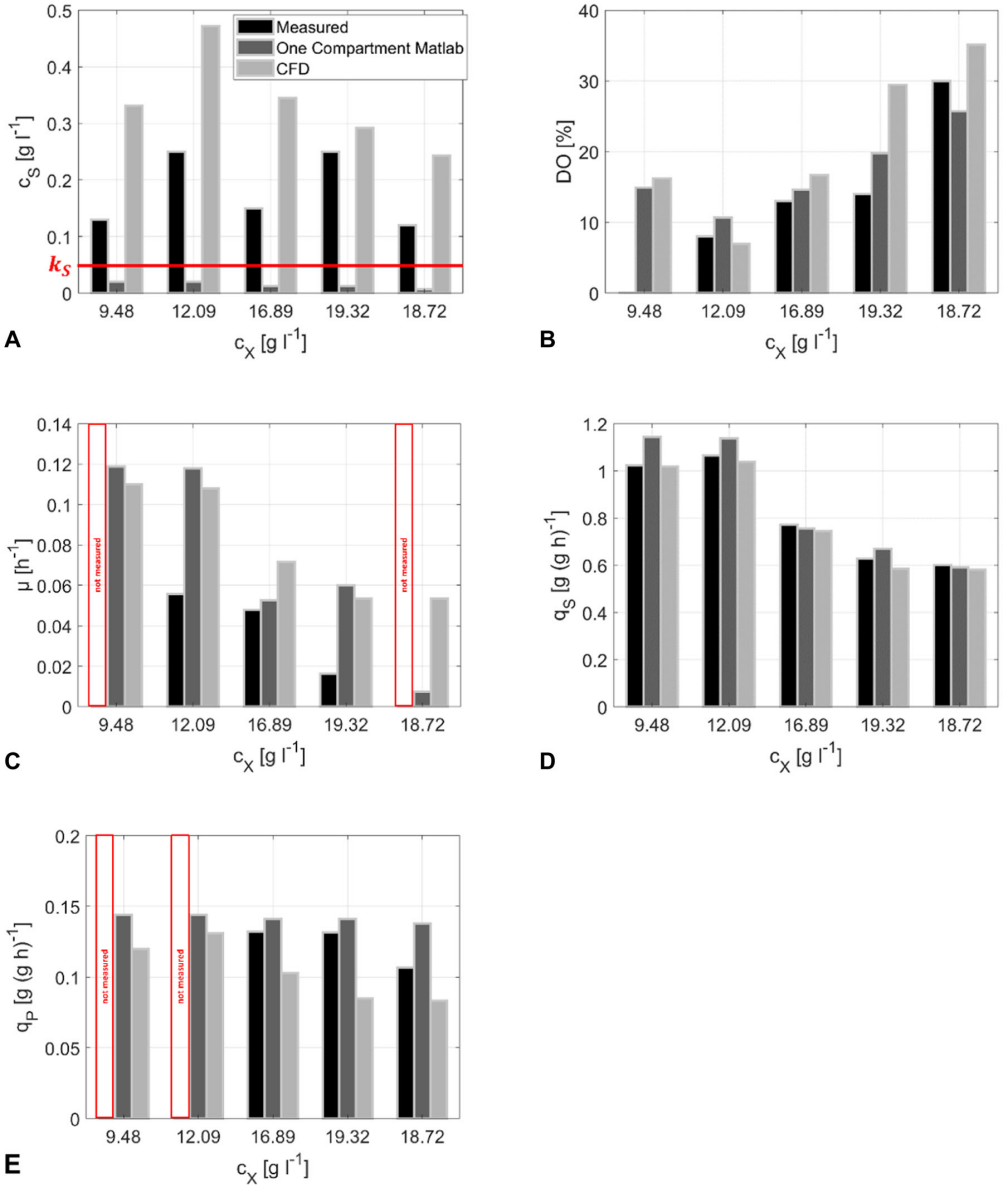
Bar plots comparing the operational states at 9, 12, 16, 20 and 24 h. Comparison with the measured values of the production run, outcomes of the one‐compartment model, and of the CFD model. Highlighted fields indicate that no experimental values were available. The growth rate from the measured values was calculated using Equation (G1) in Appendix G. (A Glucose concentration with red marked *K_S_* concentration, (B) oxygen concentration, (C) growth rate, (D) specific glucose uptake rate, and (E) specific product synthesis rate.

Motivated by the strong mixing heterogeneities, we wondered how the embedment of black‐box microbial kinetics changes the prediction quality of state variables and microbial rates compared to the simplifying one‐compartment analysis. Notably, CFD results of Figure 11 represent the average of the spatially resolved local values. In this context, Figure 11 should always be interpreted together with Figures 9 and 10, as the one‐sided view on the average value in Figure 11 may ignore the local inhomogeneities. For example, glucose and oxygen limitations occurred in large reactor zones whereas the average values does not anticipate the limitation. As indicated, one‐compartment analysis predicted very low *c*_*S*_ whereas both CFD simulations and measurements identified substrate concentrations between 0.1 and 0.5 g L^−1^. Interestingly, the latter are larger than *k*_*S*_ while one‐compartment values are not. The system exhibits a spatial limitation even though the average substrate concentration is above the half‐velocity constants. This information is missing in the one compartment model. It compensates for this by falsely predicting a spatially uniform substrate limitation estimating glucose levels below the uptake affinity.

Regarding dissolved oxygen (DO) most of the model predictions fairly well represented measured values which supports the choice of the Frossling approach for modeling oxygen transport. The largest overestimation is found for the highest biomass concentration. As all DO levels are much higher than the oxygen affinity of about 1.3% (Table 1) the conclusion might be drawn that the culture is not oxygen limited at all. However, this is misleading and caused by the simplifying one‐compartmented analysis. Figure 9A outlines that DO levels are indeed limiting in the left side of the bioreactor reflecting high metabolic activities because of locally high supply of oxygen and glucose. The latter is very limited in the right zone which reduces *q*_*S*_. As the model prioritizes carbon drain into product, biomass formation is very much hampered too, leading to low oxygen demands in the right zone of the bioreactor. As a consequence, the models fairly well predict *q*_*S*_, also manages to describe *q*_*p*_ but show highest discrepancies regarding μ. Apparently, levelling spatial variations may bias the conclusion very much.

### 3.4 In Silico Analysis of Second Feeds

**Figure 12:**
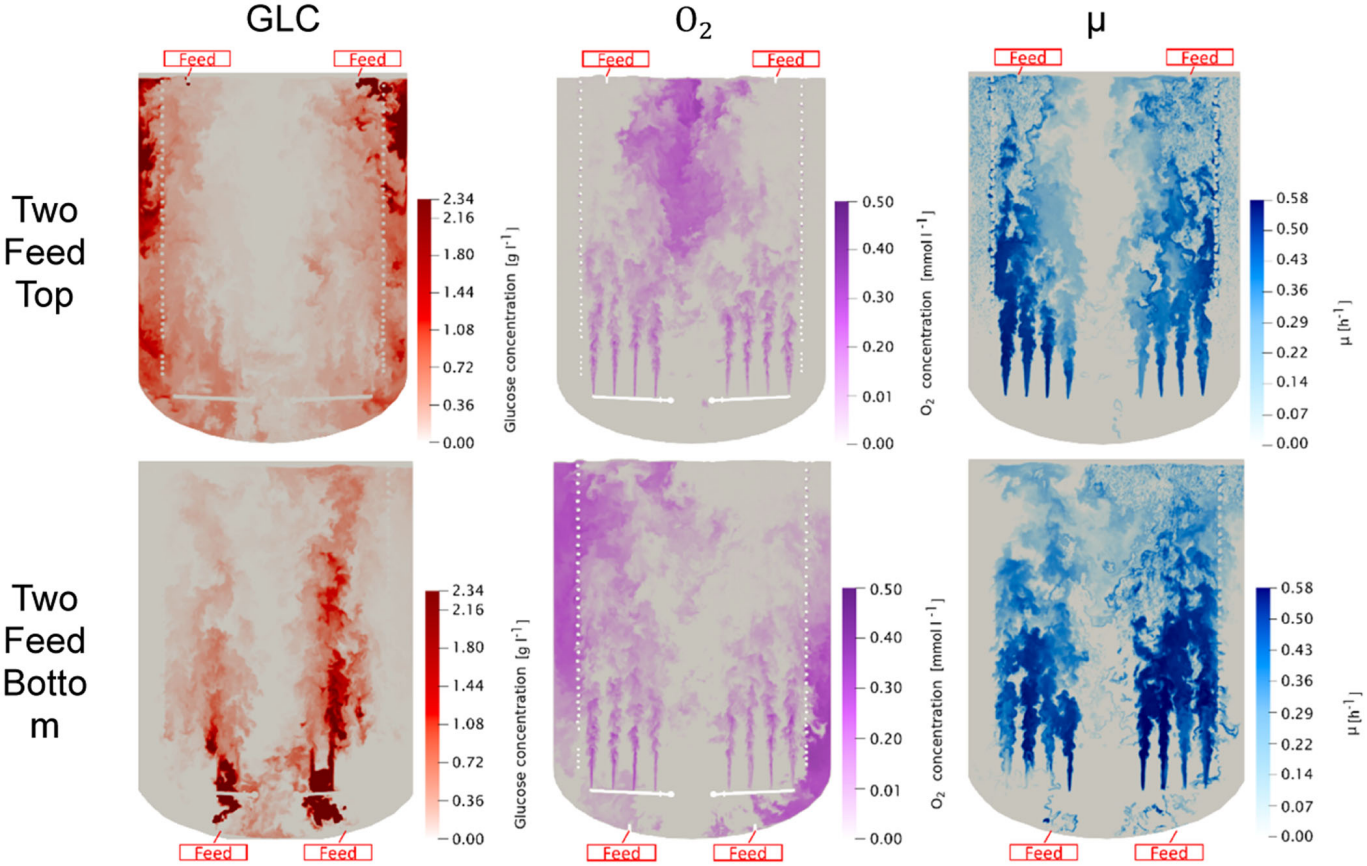
Contour plots of glucose, oxygen and growth rate on a plane intersecting the reactor's central vertical plane. The CFD simulation was calculated with process parameters according to fermentation time 4 with the maximum biomass concentration of 19.32 g l^−1^ after a fermentation of 20 h. In the first row, there are two feeding points situated at the top of the reactor. In the second row, two feeding points are positioned at the bottom of the reactor. Position of feeding inlets are shown.

Apparently, the current bioreactor setting limits glucose supply in the right zone which in turn hampers the space‐time yield. Consequently, second feed spots were considered at the top and at the bottom as shown in Figure 12. The fermentation time with highest biomass concentration is studied. Note that the sum of the feed rates always equals the rate of the single feed of the previous setting to allow comparability of the results.

The installation of a second feed at the top replaced the former bisectional left‐right difference by a left‐right reflection. Glucose was injected into the downcomer on both sides and further distributed throughout the reactor via the sparged air. The observation aligns with findings of (Larsson et al. 1996) who demonstrated the impact of feed positions (top or bottom) on the creation of substrate gradients. Now, the entire upriser zone showed rather depleted glucose concentrations whereas alleviated dissolved oxygen was observed in the zone of circulation cells.

Injecting glucose at the bottom below the air nozzles (Figure 12) led to quick mixing as the substrate benefits from the high local energy dissipation at the spargers. When the fluid entered the downcomer glucose, is already depleted leading to growth limitation and increased oxygen levels in this zone. Note, this is opposite to the scenario of top feeding.

Summarizing, top and bottom feeding with two injection points led to reduced substrate gradients. Although cellular performance remained limited by oxygen and glucose supply, the question was studied whether the implementation of a second feed already improved bioreactor performance.

**Figure 13:**
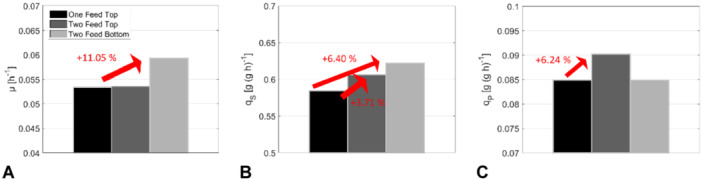
Comparison of kinetic process performance parameters for scenarios with a single feeding point at the top, two feeding points at the top, and two feeding points located at the bottom. (A) Growth rate, (B) specific substrate uptake rate, and (C) specific product synthesis rate.

As depicted in Figure 13, bottom feeding increased *μ* by 11.05% while *q*_*P*_ remained. This reflects that the setting did not increase the volumetric fraction of jointly higher glucose and oxygen levels, a prerequisite for *q*_*P*_ improvements. On contrary, additional top feeding improved the volumetric oxygen and glucose availability leading *q*_*P*_ to increase by 6.24% while μ was kept. Hence, the opposite, reversed installation of spargers and feeds minimizes axial gradients. Interestingly enough the conclusion could only be drawn by studying the spatial gradients inside the large‐scale bioreactor. This emphasizes the importance of such simulation tools for process design.

Apparently, the current design options only represent a small fraction of meaningful modifications that may be performed for optimizing the bioreactor performance. As a guiding principle, concentration gradient should be minimized to reduce cellular stress (Schweder et al. 1999; Lara et al. 2006; Löffler et al. 2016). Such studies should be accompanied by microbial lifeline analysis that study cellular experiences and response while floating inside the bioreactor (Lapin, Müller, and Reuss 2004; Haringa et al. 2016; Haringa et al. 2017; Kuschel, Siebler, and Takors 2017; Siebler et al. 2019; Puiman et al. 2023). However, this was beyond the scope of the current analysis.

## 4 Conclusion

In this work, a spatially high‐resolution CFD model was developed based on real large‐scale fermentation data. The integration of individual bubble trajectory calculations and a customized black box cell model, coupled with the fluid phase, unveiled the substantial potential for fully exploiting the production capacities. The in‐depth analysis of hydrodynamics and mass transfer revealed zones of upriser, downcomer, and circulation cells that determine the performance of the bioreactor. Highly nonlinear relationships between hydrodynamics, mass transfer including bubble dynamics, and microbial performance are elucidated that render the use of simulation tools enabling spatial resolution of said effects a *conditio sine qua non* for the design of large‐scale bioreactors.

Except from being a most valuable basis for further optimizing the performance of the current large‐scale bubble column, the study also pinpoints to remaining research needs. The currently applied Frossling approach, an example of the film‐theory, turned out to be a good choice for this setting but may be replaced by more‐sophisticated “Higbie”‐like approaches in other applications. Models correctly predicting bubble dynamics are key for the design of large‐scale bubble columns. Despite the merits of the currently applied model for bubble breakage, the equivalent for describing bubble coalescence in fermentation conditions would improve model predictions further. Moreover, sophisticated microbial models may be embedded in the CFD to provide more detailed readout of cellular responses. The latter may be well linked with lifeline studies to qualify the bioreactor with ‘the eyes of the microbe’, i.e. to design optimally with the microbial needs in mind.

## Author Contributions

**Yannic Mast:** conceptualization, execution of simulation, data acquisition, data analysis and interpretation, manuscript writing. **Adel Ghaderi:** conceptualization, manuscript editing and revision, final approval. **Ralf Takors:** supervision, conceptualization, funding acquisition, manuscript writing, editing and revision, final approval.

## Nomenclature

$c_{O_{2}}$
: oxygen concentration, mol L^−1^
$c_{O_{2}}^{*}$
: saturation oxygen concentration, mol L^−1^
$c_{P}$
: product concentration, mol L^−1^
$c_{S}$
: substrate concentration, mol L^−1^
$c_{S,F}$
: substrate feed concentration, mol L^−1^
$c_{X}$
: biomass concentration, mol L^−1^
$C_{s}$
: Smagorinsky coefficient
$D_{O_{2},L}$
: oxygen diffusion coefficient, m^2^ s^−1^
*d*
: bubble diameter, m
*d*_*32*_
: Sauter bubble diameter, m
$F_{\mathrm{in}}^{\mathrm{liq}}$
: feeding rate, L h^−1^
f
: probability density function
*g*
: gravity constant, m s^−2^
H
: Henry constant, Pa mol L^−1^
*h*
: height, m
*k*_*L*_
: liquid side mass transfer coefficient, m s^−1^
*k*_*L*_ *A*
: mass transfer of lumped bubbles, m³ s^−1^
*k*_*L*_ *a*
: integral volumetric mass transfer coefficient, s^−1^
*k*_*o2*_
: Half‐velocity constant oxygen, g L^−1^
*k*_*s*_
: Half‐velocity constant substrate, g L^−1^
*m*_*P*_
: non growth‐related product synthesis, h^−1^
*m*_*S*_
: maintenance rate, h^−1^
*P*_*head*_
: headspace pressure, Pa
Q(f,f)
: collision operator
$q_{O_{2}}$
: oxygen uptake rate, mol L^−1^ s^−1^
$q_{P}$
: total product synthesis rate, mol L^−1^ s^−1^
$q_{P,\mathrm{non}-\mathrm{growth}}$
: nongrowth‐related product synthesis rate, mol L^−1^ s^−1^
$q_{S}$
: total substrate uptake rate, mol L^−1^ s^−1^
$q_{S,\mathrm{growth}}$
: substrate uptake rate for growth, mol L^−1^ s^−1^
$q_{S,\mathrm{prod}+\mathrm{growth}}$
: substrate uptake rate for growth and product, mol L^−1^ s^−1^
$q_{S,\max}$
: maximum substrate uptake rate, mol L^−1^ s^−1^
${r_{O}}_{2}$
: oxygen mass transfer rate mol s^−1^
*t*
: time, s
$u_{\mathrm{slip}}$
: slip velocity, m s^−1^
*V*
: volume, m^3^
$\vec{v}$
: particle velocity, m s^−1^
$Y_{\mathrm{PS}}^{\mathrm{fit}}$
: fitted selectivity constant
$Y_{{\text{XO}}_{2}}$
: biomass oxygen yield
$Y_{\mathrm{XP}}^{\mathrm{fit}}$
: fitted biomass product yield
$Y_{\mathrm{XS}}^{\mathrm{fit}}$
: fitted biomass substrate yield
*y*
: volume fraction oxygen

### 1 Greek

ε
: turbulent kinetic dissipation rate, W m^−3^
$\theta_{x}$
: mixing time to level x, s
µ
: growth rate, h^−1^
${µ}_{\max}\;$
: maximum growth rate, h^−1^
$\rho_{L}$
: liquid density, kg m^−3^
σ
: surface tension, N m^−1^
ν
: viscosity, m² s^−1^

## Figures and Tables

**Figure A1:**
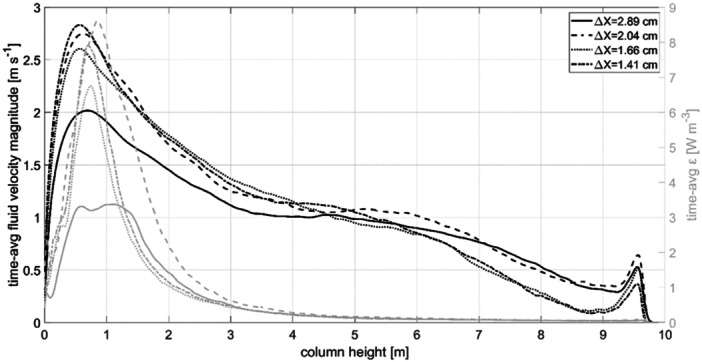
Mesh independence study respectively with 4 different mesh densities. Compared is the fluid velocity magnitude and the time averaged turbulent dissipation rate on a vertical line directly above of an air injection point.

**Figure C1:**
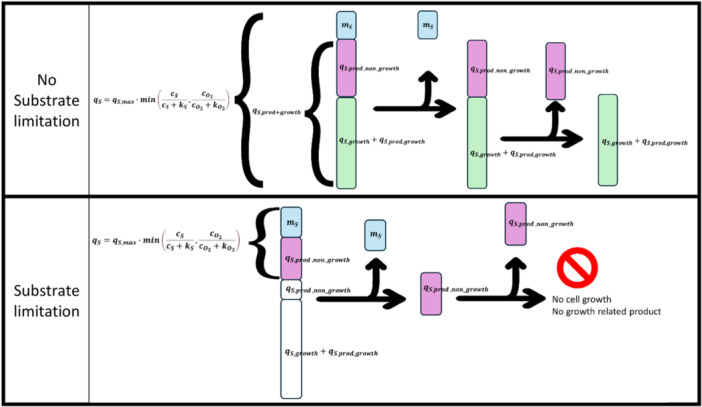
Visualization of the cell model mechanism: In case of no substrate limitation, maintenance ($m_{s}$), product synthesis ($q_{S,\mathrm{prod}})$ and cell growth ($q_{S,\mathrm{grwoth}}$) are fully supported. Under substrate limitation, priority is given first to maintenance ($m_{s}$), followed by nongrowth‐related product synthesis ($q_{S,\mathrm{prod},\mathrm{non\_growth}}$). Since the available substrate is fully utilized, cell growth and growth‐related product synthesis $(q_{S,\mathrm{prod},\mathrm{growth}}$) do not occur.

**Figure E1:**
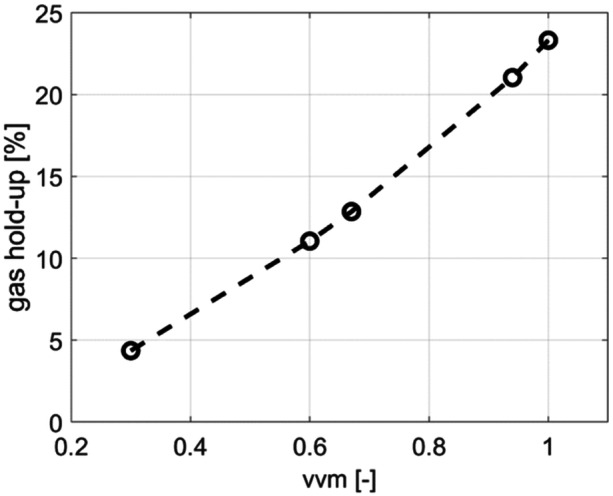
Dependency of the gas hold‐up inside the reactor for different air injection rates.

**Figure F1:**
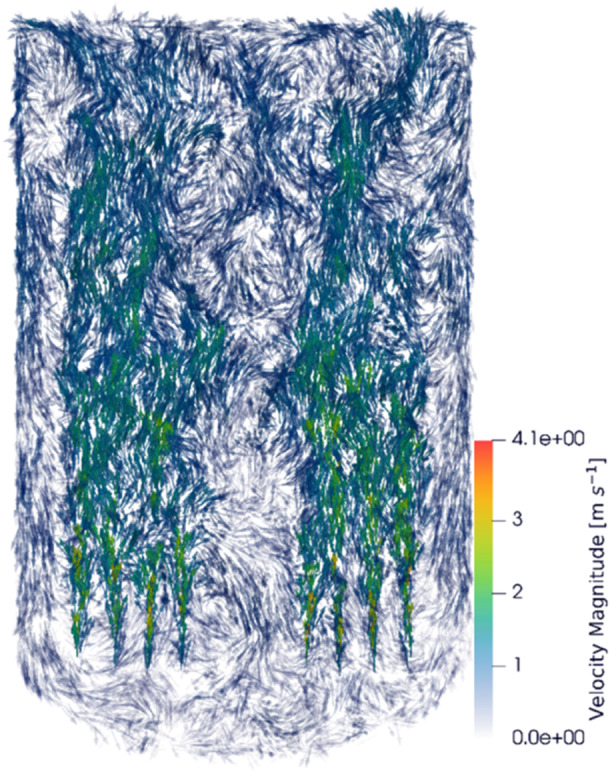
Velocity magnitude vector field on a plane intersecting the reactor's central vertical plane. The reactor is filled entirely and operated at 1 vvm.

**Figure G1:**
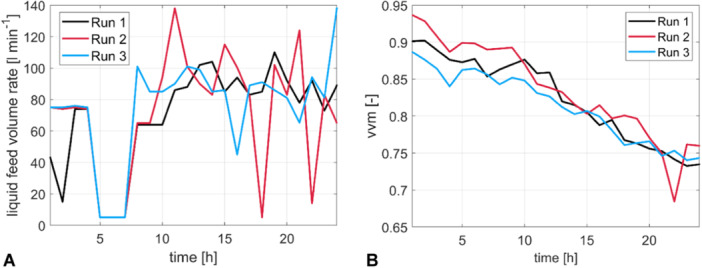
Feeding conditions over time for the three different runs. (A) Liquid feed volume rate. (B) Air volume injection rate.

## Data Availability

Data sharing not applicable to this article as no data sets were generated or analyzed during the current study.

## Appendix A

Following an assessment of a mesh independence study encompassing four distinct mesh densities, the ultimate grid size was established at ∆ *X* = 1.66 cm. The velocity magnitude exhibited a slight alteration relative to the subsequent finer grid. A coarser grid deviates from the established pattern for heights exceeding 8 m. Furthermore, the estimation of turbulent kinetic energy proves to be either overestimated or underestimated for the coarser mesh. Subsequently, the intermediate mesh was deemed sufficiently accurate, providing a balance between precision and computational efficiency.

Figure A1

## Appendix B

Figure B1

## Appendix C

Figure C1

## Appendix D

Figure D1

## Appendix E

Figure E1

## Appendix F

Figure F1

## Appendix G

Figure G1

The growth rate of the measured values from the fermentation was calculated using Equation (G1).

(G1)
$$\mu_{t}=\ln\left(\frac{c_{X,t-1}∙V_{\mathrm{reactor},t-1}}{c_{X,t+1}∙V_{\mathrm{reactor},t+1}}\right)∙\frac{1}{∆t}.$$
